# A Decrease in the Staminode-Mediated Visitor Screening Mechanism in Response to Nectar Robbers Positively Affects Reproduction in *Delphinium caeruleum* Jacq. ex Camb. (Ranunculaceae)

**DOI:** 10.3390/biology11081203

**Published:** 2022-08-11

**Authors:** Qinzheng Hou, Taihong Wang, Guang Yang, Wenjuan Shao, Wenrui Min, Yuqin Zhong

**Affiliations:** College of Life Sciences, Northwest Normal University, Lanzhou 730070, China

**Keywords:** *Delphinium caeruleum*, nectar robbing, positive effects, staminode operative strength, reproductive fitness

## Abstract

**Simple Summary:**

Nectar robbers frequently have direct or indirect negative effects on plant reproductive success. However, nectar robbers can also indirectly contribute to the reproductive success of plants in some cases. The negative effects of nectar robbing on plant reproductive success have been widely reported, but the reasons for possible positive effects demand further investigation. Hence, our study was designed to assess the effects of nectar robbers on the reproductive success of *Delphinium caeruleum*. This will facilitate an understanding of the mutualism between plants and their visitors.

**Abstract:**

Nectar-robbing insects, which are frequently described as cheaters in plant–pollinator mutualisms, may affect plant reproductive fitness by obtaining nectar rewards without providing pollination services. The negative effects of nectar robbing on plant reproductive success have been widely reported, but the reasons for possible positive effects demand further investigation. The goal of the study was to evaluate the effects of nectar robbing on the reproductive success of *Delphinium caeruleum*. Two staminodes cover the stamens and pistils in the flowers of *D. caeruleum*, forming a “double door” type of structure that compels pollinators to physically manipulate the staminodes to access the sex organs. In order to explore whether the operative strength required to open the staminodes is affected by actions associated with nectar robbing, we set up five different treatment groups: no nectar robbing, natural nectar robbing, artificial nectar robbing, hole making, and nectar removal. A biological tension sensor was used to measure the operative strength required to open the staminodes in the flowers. We also assessed the effect of nectar robbing on the flower-visiting behavior of pollinators and the effect of nectar robbing on reproductive fitness by the flower. The results showed that the operative strength needed to open staminodes was reduced by nectar robbers but not by artificial nectar robbing, hole making, or nectar removal. The flowers’ continuous visitation rate and visitation frequency by pollinators decreased significantly in robbed flowers. Both the pollen export and pollen deposition in naturally robbed flowers were significantly higher than those in nonrobbed flowers. Our results demonstrate that nectar robbers play an indirect positive role in the reproductive fitness of *D. caeruleum* flowers by reducing the operative strength of staminodes to promote pollen transfer. The reduction in operative strength of staminodes might be an adaptive mechanism that responds to nectar robbing.

## 1. Introduction

Nectar robbing is a common phenomenon in plants pollinated by insects or birds [1]. Due to a morphological mismatch between the nectar robber and the visited flower, the flower has a tubular corolla or nectar spur that is too long for the nectar robber to extract nectar legitimately [2,3], so the nectar robber extracts nectar by piercing the flower or nectar spur so that the nectar robbers visit the flowers without directly contributing to pollination [4]. According to previous studies, nectar robbers are extensively distributed geographically and are represented by a variety of species, commonly insects, among which there is a high representation of coleopteran and hymenopteran species [5,6]. Compared with the very large number of studies on the interaction between flowers and pollinators, the interaction between flowers and nectar robbers has received little attention. However, the behavior of nectar robbers may have significant evolutionary and ecological consequences on the plant populations that they target [7,8].

Nectar robbers frequently have direct or indirect negative effects on plant reproductive success. For example, the unusual foraging behavior of nectar robbers can seriously damage sex organs, resulting in robbed flowers dropping off from the plant or having a shortened life span, either of which would have direct negative effects on the reproductive fitness of the plant [9,10], whereas indirect negative effects may result from changes to the behaviors of legitimate pollinators. For instance, nectar robbers reduce the availability of nectar volume, which may then decrease the visitation frequency or visitation time of legitimate pollinators [11,12], reducing pollen export, pollen deposition, and seed set. In addition, nectar robbers of some plant species influence the behavior of legitimate pollinators, who shift to nectar robbing, a response that has indirect negative effects on the reproductive fitness of plants [12,13,14,15].

However, nectar robbers can also indirectly contribute to the reproductive success of plants in some cases. For example, corolla abscission in *Symphytum officinale* triggered by nectar robbers positively affects reproduction by enhancing self-pollination [16]. In addition, legitimate pollinators are forced to fly longer distances to visit flowers of other populations of the plant species due to reduced nectar volume availability as a result of nectar robbing, hence increasing genetic variability through increasing pollen flow and outcrossing, effects that are beneficial to the reproduction (and survival) of plant populations in the long run [17]. Previous studies have reported neutral effects of nectar robbing on the reproductive success of host plant reproduction in some flowering plant species [18,19,20]. However, the causes of negative and neutral effects can be readily detected, whereas the reasons for positive effects of nectar robbing on reproductive success need further investigation.

*Delphinium caeruleum* has two purple–blue petaloid staminodes at the center of the flower, which cover the stamens and pistils [21,22], forming a “double door” type of structure. Stamens are often called staminodes in angiosperms when they do not produce fertile pollen and cannot disperse pollen to perform their male functions after they are fully developed. The appearance of staminodes and the acquisition of their new nonmale organ functions, such as forming a barrier to prevent selfing, providing food reward, enhancing visual attraction, and producing odor to attract pollinators, are the result of long-term natural selection in the process of evolution [23,24]. According to our preliminary observations, legitimate pollinators have to open the “double-door” structure (staminodes) to visit flowers, and these pollinators make contact with the anthers and stigma below the staminodes to achieve pollination. Previous studies have reported that movable parts are found in flowers that have to be actively manipulated by insects for pollination to take place. For example, a visitor screening process of flower-visiting insects was found in *Cornus canadensis*, which screens out less forceful visitors, allowing only those with the strength to trigger the pollen catapult to pollinate it [25]. Córdoba and Cocucci called the mechanical strength needed to forcibly open such a floral mechanism the “operative strength” [26]. In our preliminary field observations, a range of flower-visiting insect species was found at the experimental site, but legitimate pollinators of *D. caeruleum* were only one species. Therefore, we propose that staminodes have the function of visitor screening, which screens out weaker insects. Previous studies have shown that in some flowers with visitor screening traits, the screening mechanism will prompt visitors that do not have the strength to achieve pollen export and dispersal to rob nectar. For example, visitor insect species are more likely to steal nectar from flowers when they cannot efficiently extract nectar from longer corollas by legitimate means [27]. Preliminary field observations indicated that there was a high proportion of nectar robber insects among visitors to *D*. *caeruleum* inflorescences, which may have been caused by the staminode-based screening mechanism. Nectar robbers of *D*. *caeruleum*, which make a hole in the nectar spur to steal the nectar without providing the pollination service, may have a negative impact on this plant’s reproductive success. Combined with the double-door staminode structure of *D*. *caeruleum*, we hypothesized that the behavior of nectar robbers might reduce the screening criteria of staminodes and then realize reproductive assurance. The aim of this research is to test these hypotheses for *D*. *caeruleum* by addressing the following questions: (1) what kind of effect does nectar robbing have on the reproductive fitness of *D. caeruleum*? (2) does nectar robbing lower the operative strength required to open staminodes? and (3), if so, will lowering the operative strength required to open staminodes improve the reproductive fitness of *D*. *caeruleum*, and, if so, how?

## 2. Materials and Methods

### 2.1. Study Species and Site

*D. caeruleum* is a perennial flowering plant from the family Ranunculaceae, for which the flowering period runs from July to September. Each flower of *D. caeruleum* on the corymbose inflorescence contains five purple–blue sepals and two petals, the latter ending with an extension to form a nectar spur within the sepal spur. Two blue staminodes with yellow barbate are located in the center of the flower, and the stamens and pistils are located at the base of the two staminodes [21]. The mating system of *D. caeruleum* is an example of obligate xenogamy [22].

The study was conducted from July to August 2021 in the Tianzhu Alpine Grassland Ecosystem test station in Wuwei City, China. The average annual temperature of the test station is 2 °C, and the average annual rainfall is 270.33 mm. The temperature range during the sampling period (August) was 4–29 °C, and the rainfall was 43.0 mm.

### 2.2. Effect of Nectar Robbing on Behavior of Visitors

In this part of the study, we focused on visitor insects to understand whether nectar robbing affected the behavior of visitors. To test whether nectar robbing led to the altered behavior of visitors, we conducted surveys in the field between 09:00 a.m. and 18:00 p.m. for 5 days (45 h in total). During the surveys, we artificially established two groups: (1) nectar-robbed flowers (selected flowers that had been naturally robbed of nectar by nectar robbers) and (2) nectar nonrobbed flowers (selected flowers that had not been robbed of nectar by nectar robbers by using a piece of clear cellophane tape to cover the nectar spurs to prevent entry by nectar robbers). Fifteen plants were assigned to each of the two treatments. We observed and recorded the foraging behavior, the flower handling time, visitation frequency of visitors, and continuous visitation rate. Insect specimens were collected in specimen boxes for later identification.

### 2.3. Biomechanical Studies

**Figure 1:**
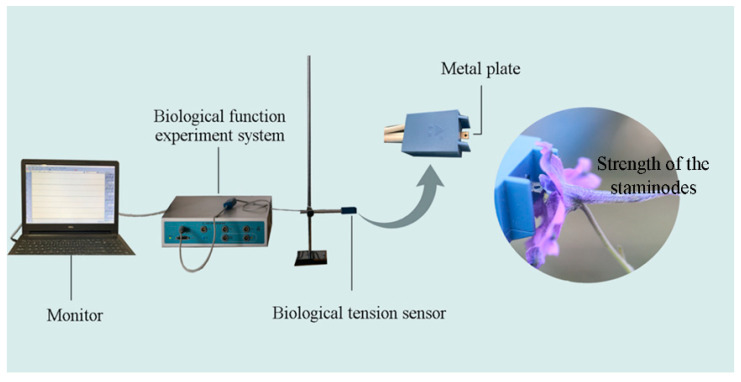
FT-102 biological tension sensor for the measurement of operative strength necessary to open the staminodes of *Delphinium caeruleum*.

We used the FT-102 biological tension sensor (Techman Soft, Chengdu, China) to measure the operative strength needed to open staminodes in attached flowers by artificially pressing the staminodes to simulate the downward pressing movement of legitimate pollinators in order to make contact with the anthers (Figure 1). We randomly selected fresh flower buds about to open from different inflorescences and used mesh bags to deny access to visitors. The next day, we examined the bagged buds and removed any unopened buds to ensure a consistent stage of florescence. The florescence period of *D. caeruleum* is 7 days in total, with the first 5 days being the male phase and the last 2 days being the female phase. In order to explore whether nectar robbing affects the operative strength needed to open staminodes, five different treatments were carried out for 7 days (each treatment group consisted of 30 new flowers each day): (1) natural nectar robbing; (2) artificial nectar robbing: we made a hole in the nectar spur to simulate the behavior of nectar robbers, then removed the nectar using a 2 mL capillary micropipette; (3) hole making: we made a hole in the nectar spur to simulate the behavior of nectar robbers but did not remove the nectar; (4) nectar removal: we used a 2 mL capillary micropipette to enter the spur from the corolla mouth to remove nectar, without making a hole; and (5) nectar nonrobbing (control): nectar robbers were denied access to the nectar spurs by using a piece of clear cellophane tape to cover the nectar spurs. Two hours after each treatment, we measured the in situ operative strength (expressed in mN) of staminodes of individual attached flowers using the biological tension sensor, with three measurements taken on each flower.

More than 1000 flowers were needed in the five treatment groups in total over the 7 days. To take into consideration the differences in temperature and light within a day, we divided the 30 flowers in each treatment group per day into two parts, processing one in the morning and one in the afternoon. For the natural nectar-robbing treatment group, as the visit of the nectar robbers was uncontrollable, we provided 50 flowers a day for the nectar robbers to visit per treatment group to ensure that we could obtain enough samples and then measured the operative strength necessary to press the staminodes 2 h after the initial visit of nectar robbers to each flower.

### 2.4. Effects of Nectar Robbing on Reproductive Output

To test whether plant reproductive fitness was affected by nectar robbing, we estimated male and female components of reproductive output in flowers in response to one of two treatments: nectar robbing by nectar robbers and nectar nonrobbing controls. We measured the number of pollen grains that legitimate pollinators removed (pollen export) and the number of pollen grains on the stigma (pollen deposition) during one visit, and the seed set (subsequent number of seeds produced). Then, we compared the differences between robbed flowers and nonrobbed flowers.

#### 2.4.1. Pollen Export

In the male phase of flowering, we used pollen export as a proxy for the male component of reproductive output by quantifying the number of pollen grains removed from an anther by legitimate pollinators during one visit. In order to calculate the number of pollen grains removed by legal pollinators in one visit, we calculated the average initial number of pollen grains in the anthers before the visit and subtracted the number of pollen grains remaining in the anthers after one visit by a legitimate pollinator from the average initial value. We randomly tagged 200 flower buds that were about to open and used mesh bags to separate visitors from the flowers. The next day, we examined the bagged buds and removed any unopened buds to ensure a consistent stage of flowering. Two different treatments were conducted (with 10 flowers being exposed to each treatment per day). (1) Nectar robbing: the mesh bags were removed, and the researchers waited for a natural nectar robber to take the nectar. After nectar robbing, the flowers were placed in mesh bags for 2 h, then the mesh bags were removed, and researchers waited for a legitimate pollinator to visit. After one visit by a legitimate pollinator, the apical dehiscent anther of each flower was stored in an Eppendorf tube containing 70% (*v*/*v*) ethanol, and the number of pollen grains in the anther was counted under a microscope. As the visit of the flower-visiting insect could not be controlled, we provided 20 flowers a day for the flower-visiting insect to visit per treatment group to ensure that we could obtain enough samples. (2) Nectar nonrobbing: the mesh bags were removed, and after one visit by a legitimate pollinator, the apical dehiscent anther was stored in an Eppendorf tube containing 70% (*v*/*v*) ethanol. The anther in the Eppendorf tube was used to count the number of pollen grains exported under a microscope. Because previous observations had shown that the first 5 days of florescence represented the male phase, we repeated the above two treatments with new flowers every day of the male phase.

#### 2.4.2. Pollen Deposition

In the female phase, we used pollen deposition as a proxy for the female component of reproductive output by quantifying the number of pollen grains deposited on the stigma by a legitimate pollinator during one visit. We randomly tagged 100 flower buds that were about to open and used mesh bags to prevent access to visitors. The next day, we examined the bagged buds and removed any unopened buds to ensure a consistent stage of florescence. Two different treatments were conducted (each represented by 30 flowers). (1) Nectar robbing: the mesh bags were removed, and researchers waited for nectar robbers to take the nectar. The flowers were then placed in mesh bags for 2 h after the nectar robbing, and then the mesh bags were removed to allow legitimate pollinators to visit. The pistil was stored in an Eppendorf tube containing 70% (*v*/*v*) ethanol after one visit by a legitimate pollinator. As the visit of the flower-visiting insect was uncontrollable, we provided 50 flowers for the flower-visiting insect to visit per treatment group to ensure that we could obtain enough samples. (2) Nectar nonrobbing: the mesh bags were removed, and the pistil was stored in an Eppendorf tube containing 70% (*v*/*v*) ethanol after one visit by a legitimate pollinator. Pollen deposition on the stigmas in the Eppendorf tubes was counted by microscopic examination after staining with lactophenol cotton blue. According to previous observations, on the sixth day of florescence, the flowers enter the female phase, and on the seventh day, the pistil matures, so we carried out the above two treatments when the pistil matures (the seventh day).

#### 2.4.3. Seed Production

We used the seed set ratio as a proxy for the overall plant reproductive output. To test whether nectar robbing affected the seed set ratio, we randomly tagged 100 flower buds that were about to open and used mesh bags to prevent access to visitors. The next day, we examined the bagged buds and removed any unopened buds to ensure a consistent florescence stage. Two different treatments were carried out (each consisting of 30 flowers): (1) Nectar nonrobbing: to prevent access to nectar robbers, we used a piece of clear cellophane tape to cover the nectar spurs. (2) Nectar robbing: flowers were exposed (no mesh bags or cellophane tape) during the first day of florescence, which provided opportunities for nectar robbers to take nectar. The following day, only those flowers where the nectar had been taken by the nectar robbers were left to ensure the consistency of the flowers in the treatment group. As the visit of the nectar robbers was uncontrollable, we provided 50 flowers for the nectar robbers to visit. After the 7-d florescence period, the flowers were placed in mesh bags to protect them and to wait for the seeds to mature. Finally, we collected all the seeds when they were mature and counted the seed set.

### 2.5. Data Analysis

The SPSS 22.0 statistical software (IBM Corp, Armonk, NY, USA) package was used to calculate and analyze the comparative test results. One-way analysis of variance (ANOVA), with the post hoc Tukey test as the multiple pairwise comparison test, was used to determine any significant differences (*p* < 0.05) in flower handling time, pollen export, pollen deposition, seed set ratio, and operative strength of staminodes under different treatments. All values are presented as mean (±standard error, SE).

## 3. Results

### 3.1. Effect of Nectar Robbing on the Behavior of Legitimate Pollinators

**Figure 2:**
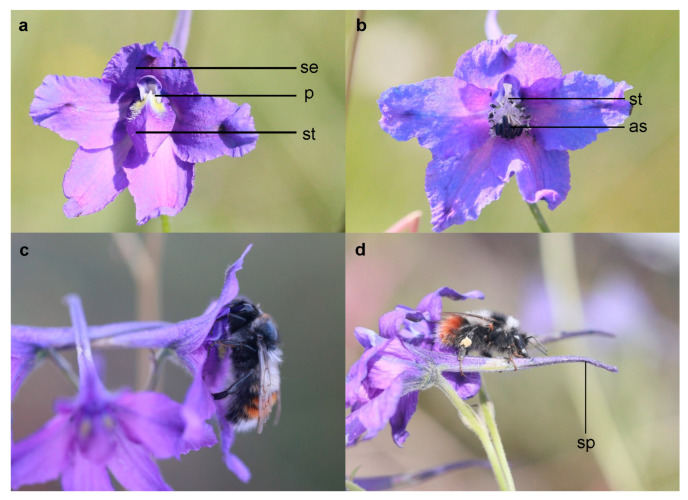
*D. caeruleum* in Tianzhu Alpine Grassland Ecosystem Test Station, Wuwei, China: (**a**) detailed floral structure of *D. caeruleum* (p, petals; se, sepals; st, staminodes); (**b**) the pistils and stamens below the staminodes (st, style; as, anther sac): (**c**) *Bumblebees rufofasciatus* legitimately visit flowers, gathering nectar through the entrance; (**d**) *Bumblebees supremus* illegitimately visit flowers, gathering nectar on spur by made a hole (sp, spur).

**Figure 3:**
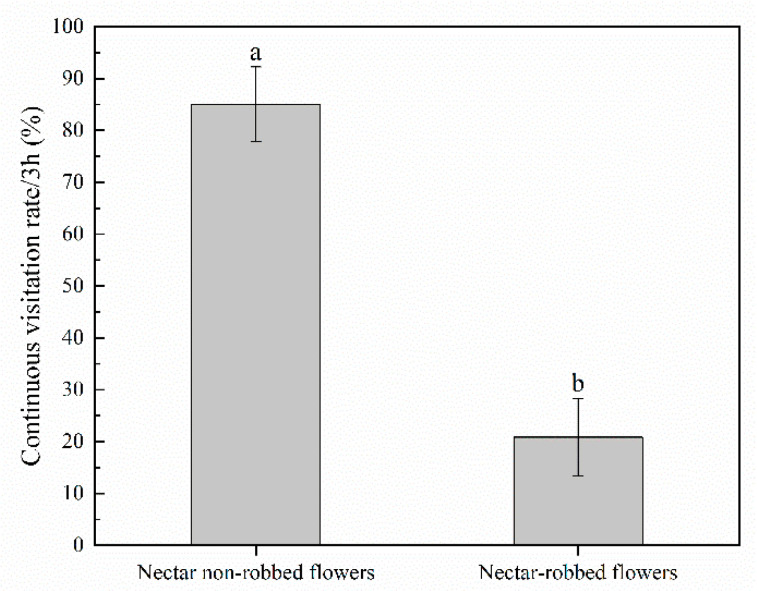
Continues visitation rate (±s.e.) of the legal pollinator (*B. rufofasciatus*) under different treatments. Different letters on items indicate significant difference at the 0.05 level.

**Figure 4:**
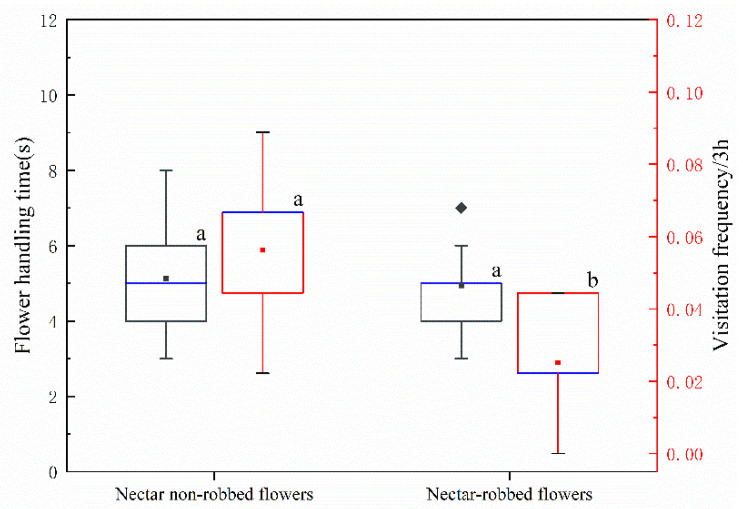
Flower handling time (±s.e.) and visitation frequency (±s.e.) of the legal pollinator (*B. rufofasciatus*) under different treatments. Black boxplots indicate the flower handling time, and red boxplots indicate the visitation frequency, showing medians, quartiles, interquartile ranges, and outliers. Different letters on items indicate significant difference at the 0.05 level.

From our observations, it was apparent that two staminodes covered the stamens and pistils of *D. caeruleum* (Figure 2a,b), which led to legitimate pollinators having to open the “double-door” structure (staminodes) by applying downward pressure to achieve pollination (Figure 2c). Two species of bumble bee visited *D.* *caeruleum* flowers in our field observations: the legitimate pollinator *Bombus rufofasciatus* Smith and the single-minded nectar robber *Bombus supremus* Morawitz (Figure 2c,d). According to our observations, nectar robbing did not affect the foraging behavior of legitimate pollinators, which never shifted to stealing nectar. However, we observed that a legitimate pollinator that visits a robbed flower will then leave that inflorescence, whereas, following a visit to a nonrobbed flower, it will visit other flowers in the same inflorescence. Our results indicate that the continuous visitation rate of legitimate pollinators to robbed flowers (20.83 ± 7.43%) was significantly lower than that of nonrobbed flowers (85.00 ± 7.24%) (Figure 3). Robbed flowers (0.0165 ± 0.0043) were visited by legitimate pollinators at a significantly lower frequency than were the nonrobbed flowers (0.0236 ± 0.0061) (Figure 4). These results indicate that the continuous visitation rate and visitation frequency were negatively affected by the phenomenon of nectar robbing. However, the flower handling time of the legitimate pollinators (*B. rufofasciatus*) was not significantly different between robbed flowers (4.94 ± 0.23 s) and nonrobbed flowers (4.94 ± 0.23 s) (Figure 4).

### 3.2. Biomechanical Studies

**Figure 5:**
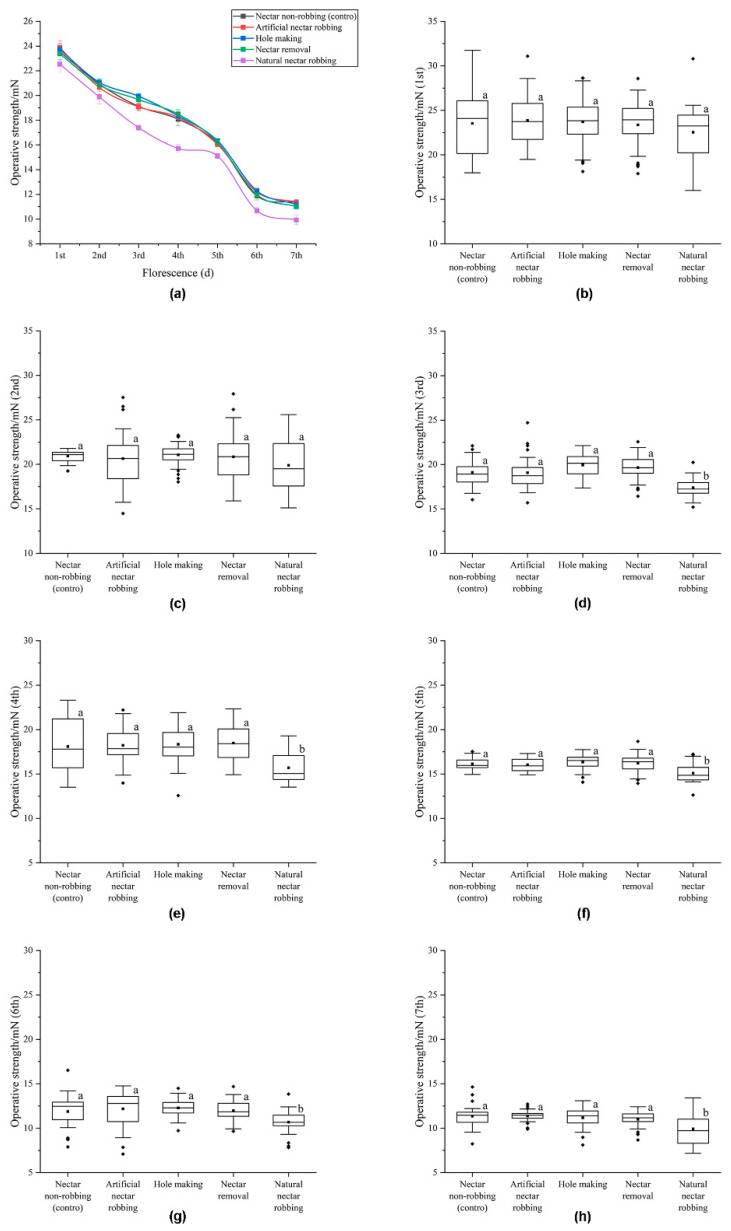
The operative strength (±s.e.) required to open staminodes of *D. caeruleum* under nectar nonrobbing, artificial nectar robbing, hole making, nectar removal, and natural nectar robbing: (**a**) dynamic change trend of operating strength required to open staminodes under five different treatments; (**b**–**h**) the operating strength required to open staminodes in 1–7 days of florescence under five different treatments. Black boxplots indicate the operative strength required to open staminodes, showing medians, quartiles, interquartile ranges, and outliers. Different letters indicate that differences are significant at *p* < 0.05.

Our results indicate that the operative strength required to open staminodes declined gradually during the 7-d florescence period of individual flowers (Figure 5a). Compared with the nectar nonrobbing treatment group, the operative strength required to open staminodes during the whole florescence period of flowers subjected to artificial nectar robbing, hole making, or nectar removal treatments were not significantly different (Figure 5b–h). Compared with the nectar nonrobbing, artificial nectar robbing, hole making, or nectar removal treatment groups, the nectar-robbing treatment group was not significantly different with respect to the operative strength required to open staminodes on the first and second days of florescence (Figure 5b–h). On the other hand, the operative strength required to open staminodes in the nectar-robbing treatment group was significantly lower than that in the nectar nonrobbing, artificial nectar robbing, hole making, or nectar removal treatment groups from the third to the seventh day of florescence (Figure 5b–h).

### 3.3. Effects of Nectar Robbing on Reproductive Success

**Figure 6:**
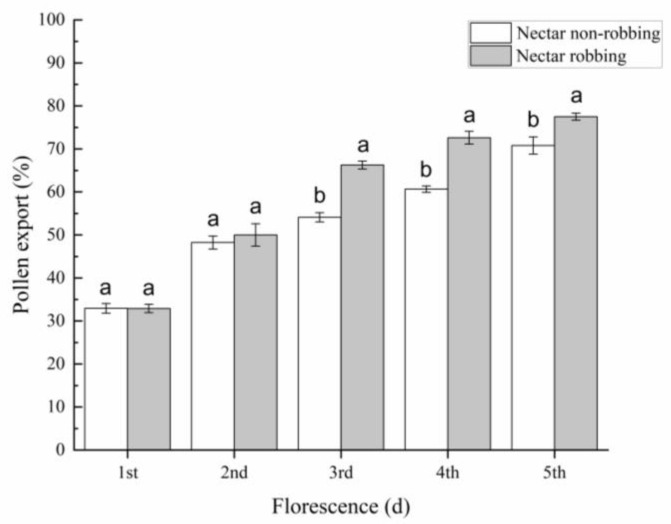
The pollen export (±s.e.) by a single visit of legal pollinator (*B. rufofasciatus*) of *D. caeruleum* from the first day to the fifth day of the male phase under nectar nonrobbing and nectar robbing. Different letters indicate that differences are significant at *p* < 0.05.

**Figure 7:**
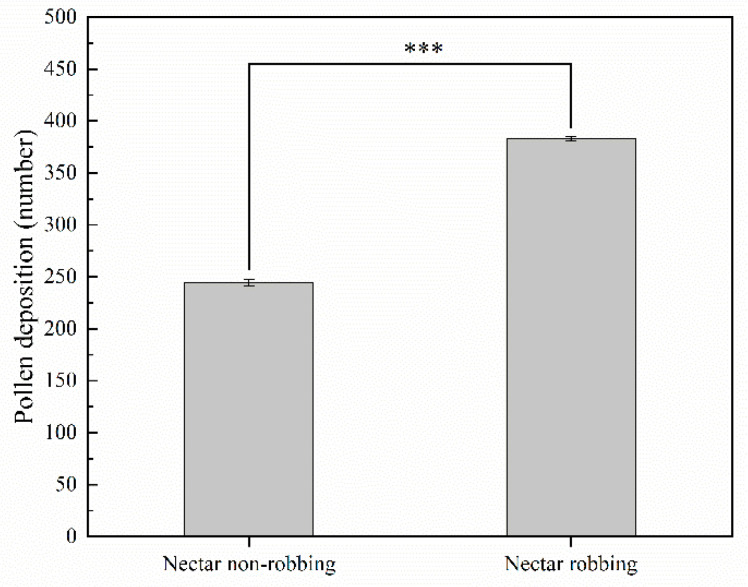
The pollen deposition (±s.e.) on stigma by a single visit of legal pollinator (*B. rufofasciatus*) of *D. caeruleum* under nectar nonrobbing and nectar robbing at female phase. Asterisks indicate a significant difference between different treatments at 0.05 level.

**Figure 8:**
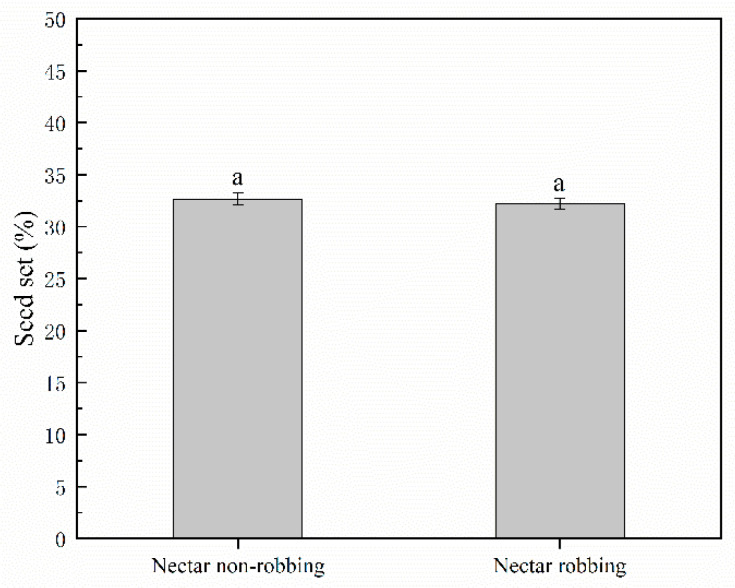
The seed set ratio (±s.e.) of *D. caeruleum* under nectar nonrobbing and nectar robbing. Identical letters indicate that differences not significant at *p* > 0.05.

Our results indicate that the pollen export achieved by a single visit by a legitimate pollinator to a nonrobbed flower (32.93 ± 1.11%; 48.24 ± 1.51%) was not significantly different from those of the robbed flower (32.90 ± 0.97%; 50.00 ± 2.61%) on the first and second days of the male phase of florescence, whereas the pollen export achieved by a single visit by a legitimate pollinator to the robbed flower (66.25 ± 0.92%; 72.61 ± 1.49%; 77.49 ± 0.82%) was significantly higher than that of the nonrobbed flower (54.10 ± 1.07%; 60.65 ± 0.75%; 70.78 ± 2.01%) on the third, fourth, and fifth day of the male phase (Figure 6). Our results showed that the pollen deposition caused by a single visit by a legitimate pollinator to the robbed flower (383.20 ± 2.10) was significantly higher than that of the nonrobbed flower (244.37 ± 3.30) during the female phase (Figure 7). With respect to seed production, our results indicated that nectar robbing did not affect overall reproductive success, with no significant difference in the seed set ratio being detected between robbed flowers (32.21 ± 0.53%) and nonrobbed flowers (32.67 ± 0.60%) (Figure 8).

## 4. Discussion

The unusual foraging behavior of nectar robbers usually damages the sex organs of the flowers or affects the behavior of legitimate pollinators, thus having direct or indirect negative effects on reproductive success [9,10,11,12]. According to our observations, the nectar robbers made a hole in the nectar spur of *D. caeruleum* to obtain nectar (primary robbing) and also used previously made holes (secondary robbing) but did not damage the sex organs (Figure 2d). Previous studies had reported that the holes made by nectar robbers might cause legitimate pollinators to act as secondary nectar robbers [12,13]. However, in the current study, the behavior of legitimate pollinators was not significantly affected, and these pollinators never became secondary nectar robbers. In addition, the flower handling time of legitimate pollinators was not significantly affected by nectar robbing (Figure 4), although we obtained evidence that the continuous visitation rate and visitation frequency of legitimate pollinators were negatively affected by nectar robbing (Figure 3 and Figure 4). The legitimate pollinator (*B*. *rufofasciatus*) was recorded at a significantly lower continuous visitation rate and visitation frequency in robbed flowers compared with nonrobbed flowers, suggesting that nectar robbing might negatively affect its pollination behavior. This might be due to nectar robbers reducing the availability of nectar volume so that legitimate pollinators are forced to fly longer distances to visit nonrobbed flowers of other populations [28]. Previous studies had shown that *D. caeruleum* has characteristics typical of herkogamy (spatial separation of stamens and pistils in the flower) and protandry, which effectively avoid the occurrence of automatic selfing [29,30,31]. Although at the level of single flowers, herkogamy and protandry can indeed avoid selfing, the continuous visitation of pollinators to an inflorescence will inevitably lead to the cross-pollination of the same plant. Our study also confirms this point: the continuous visitation rate of legitimate pollinators to flowers in the inflorescence of *D. caeruleum* was high (Figure 3). Therefore, the decrease in activity of legitimate pollinators (*B. rufofasciatus* in this study) at robbed flowers might lower the probability of reproductive success because the plant was successful in reproduction through outcrossing instead of selfing. In the meantime, nectar robbing can reduce the cross-pollination of the same plant and then promote the cross-pollination in the *D. caeruleum* population, which increases genetic variability by increasing pollen flow and outcrossing and, thus, may have an indirect positive effect on reproductive success [32,33,34].

The results from the current study indicated that the operative strength of staminodes was reduced by nectar robbers but not by artificial nectar robbing, hole making, or nectar removal, which suggests that the decrease in operative strength was not caused by mechanical damage or the decrease in nectar volume (Figure 5). Previous studies had reported a significant difference in the response of plants to mechanical damage and damage caused by phytophagous insects. The main reason for this is that the damage caused by phytophagous insects can not only cause mechanical damage to plants but also result in the secretion of herbivore-associated molecular patterns (HAMPs) during feeding or spawning [35,36]; HAMPs are signal compounds associated with phytophagous insects. Plants can recognize these HAMPs, inducing a defensive response different from that associated with simply mechanical damage [37,38]. In addition, a recent study showed that damage caused by bumble bees could promote the early flowering of plants, revealing a new interaction mode between pollinating insects and flowering plants and suggesting that bumble bees may secrete special signaling substances during the process of plant damage, to which plants respond by changing their flowering time to coordinate with bumble bees [39]. According to various evidence, therefore, we speculate that the reason for the decrease in operative strength of staminodes in response to nectar robbing was that nectar robbers might secrete a specific chemical signal during the process of stealing the nectar. *D. caeruleum* can recognize this signal to induce a response different from that achieved by mechanical damage only. This response of *D. caeruleum* to nectar robbing may be a defensive mechanism.

Our results indicate that the pollen export and deposition caused by a single visit by a legitimate pollinator significantly increased in the robbed flowers relative to the nonrobbed flowers (Figure 6 and Figure 7). According to our observations, we found that legitimate pollinators need to open the staminodes and put their heads into the corolla when visiting flowers, making contact with the stigma and anthers. In addition, our results show that the operative strength of the staminodes in robbed flowers is lower than that in nonrobbed flowers. Based on these results, we speculate that the reason for the increase in pollen export and deposition in *D. caeruleum* may be because the reduction in the operative strength of the staminodes achieves better contact between the stigma and anthers and legitimate pollinators. Nectar robbing increased pollen output and input by reducing the operative strength required to open the staminodes, which suggests that nectar robbing increases male reproductive fitness of *D. caeruleum*. Moreover, previous studies have reported that the increase in pollen deposition has a positive effect on offspring vigor [40,41]. Therefore, nectar robbing increased pollen deposition by reducing the operative strength required to open the staminodes, which suggests that nectar robbing necessarily increases the female reproductive fitness of *D. caeruleum*. On the other hand, the seed production data indicated that no significant differences were found in the seed set between robbed and nonrobbed flowers (Figure 8). The reason for this finding may be due to the fact that the average number of ovules per flower of *D. caeruleum* is 110, and the pollination efficiency of legitimate pollinators is high, so a small number of effective visits by legitimate pollinators may ensure the high level of development of ovules [42]. In conclusion, we consider that the nectar robbers have an indirect positive effect on the reproductive fitness of *D. caeruleum*.

## 5. Conclusions

In conclusion, our study indicated that neither legitimate pollinator behavior nor handling time was significantly influenced by nectar robbers, but that nectar robber action influenced the continuous visitation rate and visitation frequency of robbed flowers by legitimate pollinators, which may have potentially negative effects on reproductive fitness. However, this negative impact is overcome by the dynamic change in the operative strength of the staminodes. Our results showed that the operative strength of staminodes of *D. caeruleum* decreased significantly after the nectar robbers visited flowers, which increased male and female reproductive fitness. The decreased operative strength of staminodes may be a defense mechanism in response to nectar robbing, ensuring plant population reproductive success. However, some authors have questioned that the decrease in operative strength of staminodes instead improved male reproductive fitness, so why are staminodes necessary for the plants’ survival? Previous studies have reported that brightly colored or petaloid staminodes can attract pollinators [43,44]. Furthermore, the staminodes surrounding fertile stamens and stigmas in *Lopezia clavata* and species of the order Magnoliales play a protective role [23,45]. Our observations found that the staminodes of *D. caeruleum* were purple–blue with yellow barbate and covered its anthers and stigma tightly, which suggest that it may have functions of attracting pollinators and protecting the sex organs at the same time. However, further studies on staminodes are needed. In addition, careful experimental and observational research is still needed to uncover the subtleties in the interaction between plant species, nectar robbers, and pollinators; for example, how does *D. caeruleum* identify nectar robbers and how does the internal mechanism of operative strength of the staminodes decrease in response to nectar robbers?

## Data Availability

The data presented in this study are available on request from the corresponding author.
